# Blackfly fever and dermatitis caused by *Simulium kiritshenkoi:* a human case report in Iran

**DOI:** 10.1186/s12879-020-05070-y

**Published:** 2020-05-15

**Authors:** Farbod Tabatabaei, Sahar Azarmi, Mohammad Javad Abbaszadeh Afshar, Hamed Yarizadeh, Sina Mohtasebi

**Affiliations:** 1 School of Allied Medical Sciences, Iran University of Medical Sciences, Tehran, Iran; 2 Department of Medical Entomology and Vector Control, School of Public Health, Tehran University of Medical Sciences, Tehran, Iran; 3 Department of Medical Parasitology and Mycology, School of Public Health, Tehran University of Medical Sciences, Tehran, Iran; 4 Department of Medical Parasitology and Mycology, School of Medicine, Jiroft University of Medical Sciences, Jiroft, Iran

**Keywords:** *Simulium*, Blackfly fever, Dermatitis, Iran, Case report

## Abstract

**Background:**

Besides the considerable role of blackflies to the transmission of many disease agents, these flies considered as annoying biting pests of wildlife, livestock, poultry, and humans. There are few literature reports of blackfly fever following *Simulium spp.* This study describes a case of blackfly fever and dermatitis following blackflies numerous bites in Iran.

**Case presentation:**

The present report describes a 25-year-old man that was attacked by numerous flies while fishing and camping near Namrood river in Firuzkuh County, Tehran Province, Iran. Pruritic dermatitis with marked edema appeared mainly on the hands and legs and subsequently, the patient’s condition worsened with swollen lymph nodes, joints aching, and 40 °C fever. The patient’s clinical signs and symptoms were alleviated by injection of intramuscular Dexamethasone Phosphate (DEXADIC®) 8 mg/2 ml after 24 h.

**Conclusions:**

This study reported a human case with blackfly fever and dermatitis following numerous bites of *Simulium kiritshenkoi,* for the first time in Iran.

## Background

Blackflies or buffalo gnats (Diptera: Simuliidae) have a wide distribution around the world, consisting of more than 2300 known species in 19 genera [1]. Twenty-three species of this family have been reported from different parts of Iran [2]. Humped thorax, squat body, 11-segmented antennae and broad wings are the most prominent morphological characters of blackflies [3]. Adult females of simuliids are of medical and veterinary importance in temperate regions of the world. The most important species of these flies are in the genus *Simulium*, which act as vectors for *Onchocerca volvulus* and *Mansonella ozzardi* causing human onchocerciasis and mansonellosis, in tropical and temperate regions, respectively. Moreover, they transmit pathogenic nematodes (such as *Onchocerca gutturosa* and *Onchocerca cervicalis*), viruses causing Eastern equine encephalitis and vesicular stomatitis, protozoa (the genus *Leucocytozoon*) to livestock and birds [3, 4]. Also, blackfly bites could cause local pain, redness, dermatitis, wounds, blackfly fever, and retropharyngeal edema in humans due to a reaction to blackfly salivary compounds [4, 5]. Blackfly fever is a systemic reaction following a blackfly bite that includes headache, nausea, fever, weakness, and swollen lymph nodes [1]. In the present study, a case of blackfly fever and dermatitis following numerous blackfly bites, is described in Firuzkuh County, Tehran Province, center of Iran.

## Case presentation

**Fig. 1:**
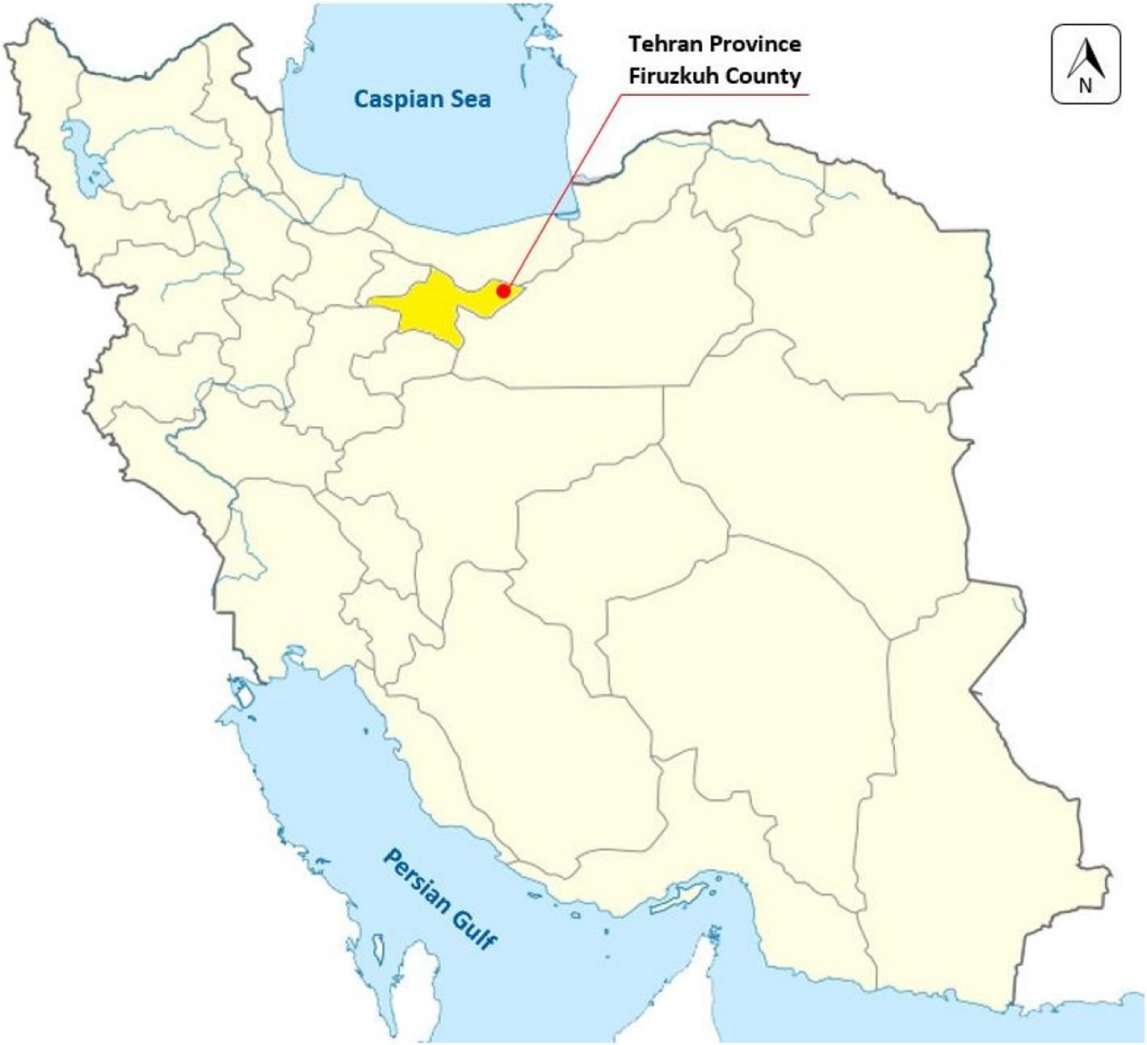
Map of Iran, Firuzkuh location in Tehran Province

**Fig. 2:**
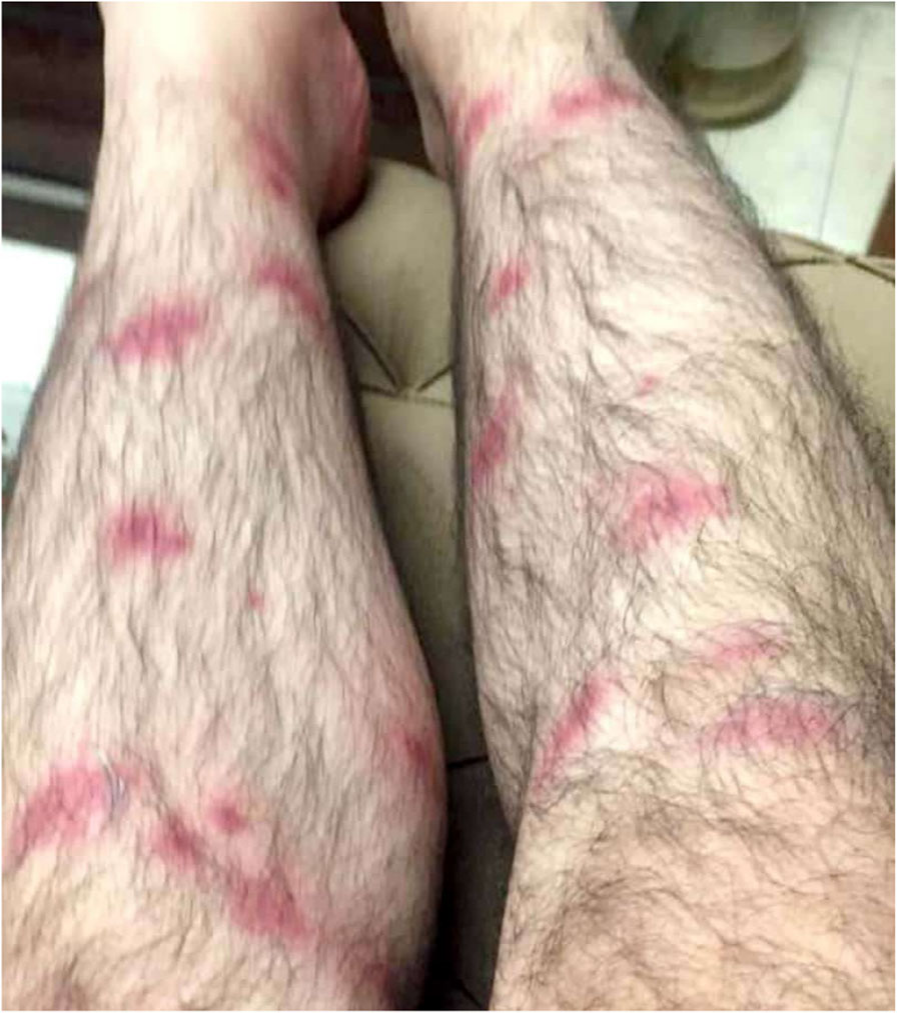
The lesions on the legs. Inflammation and redness around biting sites is visible

In September 2019, a 25-year-old man from Tehran province, while fishing and camping in Namrood river in Harandeh village, Firuzkuh County, Tehran Province, Iran (35.7550° N, 52.7724° E) (Fig. 1) was attacked by numerous *Simulium* flies at early morning. After few minutes, pruritic dermatitis with considerable edema appeared in biting sites on hands and legs (Fig. 2). In the next few hours, the patient’s condition aggravated in reactions to blackflies bites by appearing swollen lymph nodes, joints aching, and 40 °C fever. The patient’s clinical signs and symptoms were alleviated by single injection of intramuscular Dexamethasone Phosphate (DEXADIC®) 8 mg/2 ml after 24 h after referring to an infectious disease specialist. However, the pruritic lesions completely healed after 10–14 days with no further treatment. Meanwhile, the interview revealed that the patient had no history of allergic diseases and had not yet experienced such condition.

The flies were captured on the skin during blood-feeding by the patient and kept in a capped container. All captured flies were brought to the Department of Medical Parasitology, School of Public Health, Tehran University of Medical Sciences for further investigations.

**Fig. 3:**
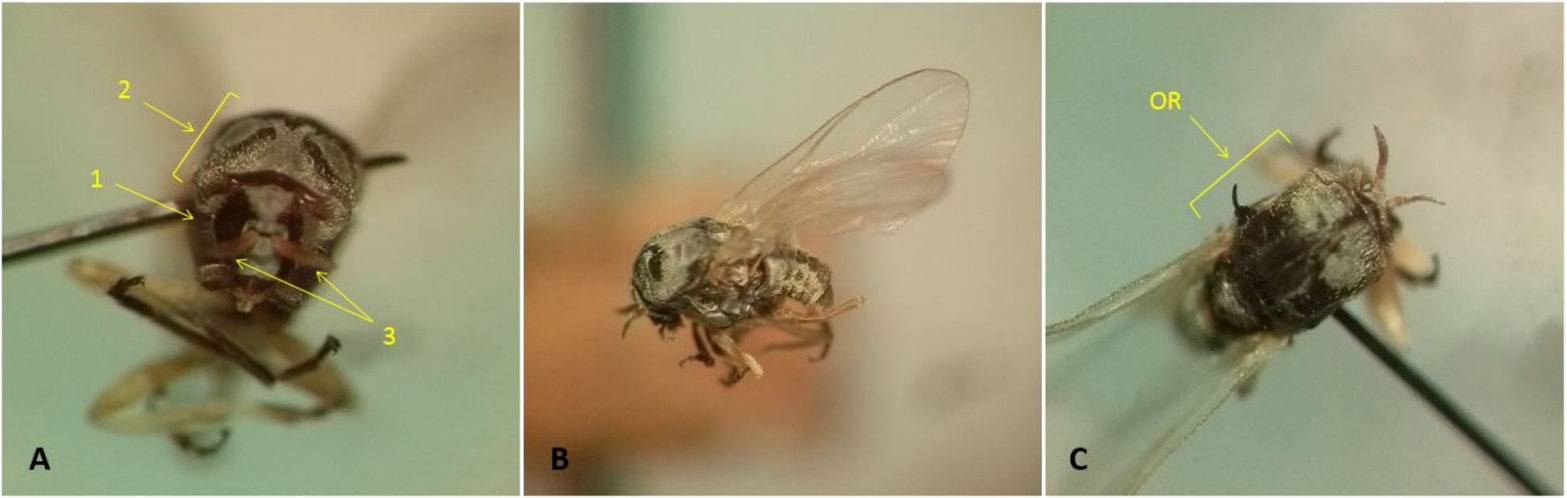
Adult female Simulium kiritshenkoi. **a**: 1. dichoptic eyes 2. Front view of scutal pattern 3. Reddish orange antennae, **b**: Lateral view, **c**: Ornatum pattern of scutum (OR)

By investigating the morphological characteristics, the collected adult blackflies were identified as the females *S. kiritshenkoi* (Fig. 3). In the female blackflies, the eyes are separated (dichoptic). While, in the males, the eyes are close together (holoptic). Tarsus of the hind leg with calcipala, no hair at the base of radius vein, two silvery grey parts at the front of the scutum, the hairy pleural membrane, strongly beak-like process on the ventral plate of the genitalia, and red or reddish-orange antennae are some morphological characters of adult male *S. kiritshenkoi*. Adult females of *S. kiritshenkoi* have a hairy pleural membrane, and calcipala on the tarsus of the hind leg. Scutum is black or dark grey-black with ornatum pattern. Antennae are orange-red [6].

## Discussion and conclusions

Harandeh village in Firuzkuh County has a cold climate and Namrood river passes through the village and many fishermen and campers visit there for fishing and camping. Blackflies are abundant in that area. Hence, the risk of blackflies bites is of great significance. In the present study, the captured flies were identified as *S. kiritshenkoi* based on available keys.

In 1970–1980 three genera, seven sub genera and 17 species of simuliides were identified from Iran and Iraq [6]. In a complementary study in 2010–2012, 23 species of blackflies were recognized and reported [2]. Previously *S. kiritshenkoi* and *Simulium caucasicum* have been reported from Namrood river which confirms our investigations [7].

In a study by Youssefi et al., a case of dermatitis caused by blackflies bites in a 32 year-old-man was reported in Lorestan Province, western Iran [8].

In the present study, blackfly fever and dermatitis were observed in the patient after *S. kiritshenkoi* bites. Blackfly fever is a systemic reaction in humans following a blackfly bite that includes headache, nausea, joints aching, high fever, weakness, and swollen lymph nodes [1].

In Japan, Watanabe described an 8-year-old boy with Kawasaki disease and retropharyngeal edema following blackflies bite [5]. Kawasaki disease, known also as mucocutaneous lymph node syndrome, causes inflammation in the walls of blood vessels throughout the body. This disease is a rare childhood disease. Rash, redness, fever, lymph nodes swelling in the neck, and irritation and inflammation of the mucous membranes are some symptoms of Kawasaki disease [9]. The Watanabe results conclude that systemic T cell activation due to a blackfly bites and high volume of salivary secretions may have resulted in the development of Kawasaki disease in patients. Numerous bites and injection of the salivary secretions lead to a variety of reactions from itching and local swelling to severe allergic reactions, blackfly fever or even anaphylactic shock and death [10].

As regards the veterinary importance of black fly, cases of severe dermal lesions and death in livestock following the mass attack of *Simulium maegaitae* have been reported in Arasbaran area in northwestern Iran [11].

Although there is no report of river blindness in Iran, a rare case of human ocular onchocerciasis caused by *Onchocercia lupi* has been reported [12]. The increasing human cases in Europe and the United States of America confirm the zoonotic role and importance of *O. lupi* parasite [13, 14].

Because of the biphasic life cycle of simuliids, including winged adults and aquatic stages, the blackfly control is extremely complex [1]. Black flies worldwide are managed primarily through the use of the bacterium *Bacillus thuringiensis* var. *israelensis,* which is aimed at the larval stage. Chemical insecticides such as permethrin are not selective and have deleterious impacts on other aquatic communities like Plecoptera and Ephemeroptera, which are considered as a vital food source for insectivorous fishes, therefore, they are used only in a few areas of the world [1, 15]. People should avoid outdoor activity during the early morning, and evening particularly in warm weather. Also, it is suggested to wear protective clothes and repellents for personal protection.

In conclusion, this study reported a human case with blackfly fever and dermatitis following *S. kiritshenkoi* bites for the first time in Iran. According to the comprehensible medical and veterinary importance of blackflies in the transmission of diseases and their direct harms, it is worthwhile to study the species of blackflies and their distribution in Iran to facilitate appropriate preventive measures.

## Data Availability

All data and materials of this article are included in the manuscript.
